# Tolerability of four‐drug antiretroviral combination therapy in primary HIV‐1 infection

**DOI:** 10.1111/hiv.13118

**Published:** 2021-05-08

**Authors:** JE Burns, W Stöhr, S Kinloch‐De Loes, J Fox, A Clarke, M Nelson, J Thornhill, A Babiker, J Frater, SL Pett, S Fidler

**Affiliations:** ^1^ Centre for Clinical Research in Infection and Sexual Health Institute for Global Health University College London London UK; ^2^ Medical Research Council Clinical Trials Unit Institute of Clinical Trials and Methodology University College London London UK; ^3^ Department of Infection and Immunity Royal Free Hospital London UK; ^4^ Institute of Immunity & Transplantation University College London London UK; ^5^ Department of Genitourinary Medicine and Infectious Diseases, Guys and St Thomas’ NHS Trust London UK; ^6^ Department of Genitourinary Medicine and Infectious Diseases NIHR Biomedical Research Centre King’s College London London UK; ^7^ Elton John Centre Brighton UK; ^8^ Department of HIV and Sexual Health Sussex University Hospital Brighton UK; ^9^ Brighton and Sussex Medical School University of Sussex Brighton UK; ^10^ Department of HIV Medicine Chelsea and Westminster Hospital Imperial College London London UK; ^11^ Department of Infectious Disease Imperial College London London UK; ^12^ NIHR Imperial Biomedical Research Centre London UK; ^13^ Nuffield Department of Medicine Oxford University Oxford UK; ^14^ Nuffield Department of Medicine Oxford NIHR Biomedical Research Centre Oxford UK

**Keywords:** adherence, antiretroviral therapy, primary HIV‐1 infection, tolerability

## Abstract

**Objectives:**

Rapid initiation of antiretroviral therapy (ART) is important for individuals with high baseline viral loads, such as in primary HIV‐1 infection (PHI). Four‐drug regimens are sometimes considered; however, data are lacking on tolerability. We aimed to evaluate the tolerability of four‐drug regimens used in the Research in Viral Eradication of HIV‐1 Reservoirs (RIVER) study.

**Methods:**

At enrolment, ART‐naïve adult participants or those newly commenced on ART were initiated or intensified to four‐drug regimens within 4 weeks of PHI. Rapid start was defined as pre‐confirmation or ≤ 7 days of confirmed diagnosis. Primary and secondary outcomes were patient‐reported adherence measured by 7‐day recall and regimen switches between enrolment and randomization, respectively.

**Results:**

Overall, 54 men were included: 72.2% were of white ethnicity, with a median age of 32 years old, 42.6% had a viral load of ≥ 100 000 HIV‐1 RNA copies/mL, and in 92.6% sex with men was the mode of acquisition of HIV‐1. Twenty (37%) started a four‐drug regimen and 34 (63%) were intensified. Rapid ART initiation occurred in 28%, 100% started in ≤ 4 weeks. By weeks 4, 12, and 24, 37.0%, 69.0%, and 94.0% were undetectable (viral load < 50 copies/mL), respectively. Adherence rates of 100% at weeks 4, 12, 22 and 24 were reported in 88.9%, 87.0%, 82.4% and 94.1% of participants, respectively. Five individuals switched to three drugs, four changed their regimen constituents, and two switched post‐randomization.

**Conclusions:**

Overall, four‐drug regimens were well tolerated and had high levels of adherence. Whilst their benefit over three‐drug regimens is lacking, our findings should provide reassurance if a temporarily intensified regimen is clinically indicated to help facilitate treatment.

## Introduction

Following the findings of the START and TEMPRANO trials in 2015 [1, 2], HIV‐1 treatment guidelines are unified in their recommendation to initiate antiretroviral therapy (ART) irrespective of CD4 count [3, 4]. There is also a consensus that the rapid initiation of ART (ideally ≤ 7days after confirmed HIV‐1 diagnosis) is feasible [5], can achieve faster virological suppression [6], minimizes the HIV‐1 reservoir [7] and subsequent immune recovery [1], and improves uptake of ART and retention of care [8]. Rapid ART initiation is particularly important for individuals with primary HIV‐1 infection (PHI) to mitigate the elevated risk of onward transmission due to very high HIV‐1 viral loads [9, 10].

Current guidelines for rapidly starting ART in PHI recommend triple ART regimens comprising a tenofovir‐based, dual nucleos(t)ide reverse transcriptase inhibitor (NRTI) backbone combined with integrase strand transfer inhibitors (INSTIs), or a boosted protease inhibitor (PI) such as darunavir/ritonavir (DRV/r) [4]. Some physicians elect to start all four components, particularly with high viral loads in PHI. The rationale for four drugs is to access the benefits of faster viral suppression seen with INSTIs [11, 12] combined with the higher genetic barrier to resistance associated with PIs [13]. This also safely negates the need to await genotype resistance and HLA‐B*5701 results.

Despite the recommendations for rapid start of ART in PHI, there is a paucity of data on the tolerability and adherence in this setting, a time when patients are dealing with the burden of a new diagnosis of HIV‐1, potentially compounded by symptoms of seroconversion. As such, our aim was to review the tolerability of four‐drug regimens in the Research in Viral Eradication of HIV‐1 Reservoirs (RIVER) trial (NCT02336074) [14].

## Methods

The RIVER trial methodology is described in the primary manuscript [14]. RIVER was conducted in the UK, during 2016–2018. At enrolment, ART‐naïve adult participants or those newly commenced on ART were initiated or intensified to four‐drug regimens within 4 weeks of PHI. They were randomized 6 months later to adjuvant ChAdV63.HIVconsv‐prime and vorinostat or to continue ART alone. The post‐randomization intervention period lasted 18 weeks. This analysis only includes participants who received a four‐drug ART regimen.

Participants were recommended a four‐drug ART regimen as per the RIVER protocol. This included daily DRV/r 800/100 mg, as per the guidance for ART initiation prior to genotype availability at the time of recruitment [3], raltegravir 400 mg twice a day to facilitate rapid viral load suppression, and a dual, tenofovir‐based NRTI backbone. For those on a three‐drug combination pre‐enrolment, intensification was proposed with raltegravir if on a PI‐based regimen or a boosted PI if on a raltegravir‐based regimen.

Our primary outcome was patient‐reported adherence measured by 7‐day recall at weeks 0, 4, 12, 22 and 24 (randomization). The 7‐day recall tool is widely used in trials conducted by the AIDS Clinical Trials Group and the International Network for Strategic Initiatives in Global HIV Trials [15]. Our secondary outcome was the number of regimen switches between enrolment and randomization.

## Results

This male cohort (*n* = 54) had a median age of 32 years [interquartile range (IQR): 29–40], 72.2% were of white ethnicity (*n* = 39), and in 92.6% (*n* = 50) sex with men was the likely mode of HIV‐1 acquisition. Median (IQR) HIV‐1 viral load was 48 295 (19 408–1 073 031) copies/mL; 42.6% (*n* = 23) had a viral load ≥ 100 000 copies/mL. The majority (*n* = 34, 63.0%) were enrolled in the study based on a recent infection test algorithm [16]; 27.7% (*n* = 15/54) started ART ≤ 7 days after a confirmed diagnosis (rapid start).

Of the 34 (63.0%) on ART pre‐enrolment, 21 received a PI‐based three‐drug regimen, three received a raltegravir‐based three‐drug regimen, and nine received a four‐drug regimen with raltegravir, boosted darunavir, tenofovir disoproxil fumarate (TDF) and emtricitabine (FTC). One participant received TDF/FTC pre‐exposure prophylaxis. The 20 (37.0%) ART‐naïve individuals at enrolment all started a four‐drug regimen (Table 1). The times from confirmed PHI diagnosis to ART initiation were ≤ 1, 1–2, 2–3 and 3–4 weeks in 14.8% (*n* = 8), 22.2% (*n* = 12), 16.7% (*n* = 9) and 33.3% (*n* = 18), respectively. Seven (13.0%) started ART before HIV‐1 diagnosis confirmation; 85.2% (*n* = 46) remained on the same four‐drug regimen from enrolment to randomization which occurred at week 24 (range 22–54). By weeks 4, 12, and 24 post‐enrolment, 37.0%, 69.0%, and 94.0% had a viral load < 50 copies/mL respectively.

**Table 1:** Summary of baseline status and antiretroviral therapy (ART) regimen combinations. Data are *n* (%) unless noted otherwise

	Men
Sample	54 (100.0)
HIV‐1 viral load at enrolment (copies/mL)	
Overall average [median (IQR)]	48 295 (19 408–1 073 031)
< 200	2 (3.7)
1000 to < 10 000	7 (13.0)
10 000 to < 100 000	22 (40.7)
100 000 to < 1 000 000	9 (16.7)
≥ 1 000 000	14 (25.9)
Confirmed PHI diagnosis to start of ART (weeks)	
Before confirmed diagnosis	7 (13)
≤1	8 (15)
1–2	12 (22)
2–3	9 (17)
3–4	18 (33)
Confirmed PHI diagnosis to four‐drug ART start (weeks)	
Before confirmed diagnosis	1 (1.8)
≤1	4 (7.4)
1–2	7 (13.0)
2–3	11 (20.4)
3–4	31 (57.4)
First ART regimen pre‐enrolment (*n* = 34)	
TDF/FTC*	1 (3)
TDF/FTC + DRV/r	16 (47)
TDF/FTC + DRV/c	5 (15)
TDF/FTC + RAL	3 (9)
TDF/FTC + DRV/r + RAL	5 (15)
TDF/FTC + DRV/c + RAL	4 (12)
First ART regimen post‐enrolment (*n* = 20)	
TDF/FTC + DRV/r + RAL	13 (65)
TDF/FTC + DRV/c + RAL	6 (30)
TDF/FTC + EFZ + RAL	1 (5)

ABC, abacavir; ART, antiretroviral therapy; DRV, darunavir; DTG, dolutegravir; EFZ, efavirenz; FTC, emtricitabine; IQR, interquartile range; TDF, tenofovir disoproxil fumarate; PHI, primary HIV‐1 infection; RAL, raltegravir; 3TC, lamivudine; /c, cobicistat‐boosted; /r, ritonavir‐boosted.

* Individual was taking pre‐exposure prophylaxis.

Adherence at weeks 4, 12, 22, and 24 (randomization) indicated 100% adherence to ART in 88.9% (*n* = 48/54), 87.0% (*n* = 47/54), 82.4% (*n* = 42/51) and 94.1% (*n* = 48/51) of participants, respectively. Three individuals withdrew or were lost to follow‐up before randomization. Five changed to a three‐drug regimen [at weeks 7 (*n* = 2), 24, 26 and 40) and three changed components of, but remained on, a four‐drug regimen (Table 2). The reasons for ART changes included adverse events (*n* = 5; Table 2), pill burden (*n* = 1), recreational drug interaction (*n* = 1) and patient choice (*n* = 1). Only two participants changed their four‐drug regimen post‐randomization – one for declining renal function and one due to nausea and vomiting.

**Table 2:** Antiretroviral therapy (ART) regimen changes

ART change	Initial regimen				Switched regimen				Switch reason	Week post‐enrolment
1	TDF	FTC	DRV/c	RAL	TDF	FTC	DRV/r	RAL	Diarrhoea	4
2	TDF	FTC	DRV/r	RAL	TDF	FTC	RAL	‐	Insomnia	7
3	TDF	FTC	DRV/r	RAL	TDF	FTC	DRV/c	‐	Scalp Alopecia	7
4*	TDF	FTC	DRV/r	RAL	ABC	3TC	DRV/r	RAL	Decreased renal function	10
5*	ABC	3TC	DRV/r	RAL	TDF	FTC	DRV/r	RAL	Vivid dreams	14
6	TDF	FTC	DRV/r	RAL	TDF	FTC	DRV/c	DTG	Patient choice	24
7	TDF	FTC	DRV/r	RAL	TDF	FTC	RAL	‐	Pill burden	24
8	TDF	FTC	DRV/r	RAL	TDF	FTC	RAL	‐	Decreased renal function	26
9	TDF	FTC	DRV/r	RAL	FTC	DRV/r	RAL	‐	Concerns about recreational drug interactions	40
Post‐randomization										
1^†^	TDF	FTC	EFV	RAL	TDF	FTC	DRV/r	RAL	Nausea and vomiting	37
2	TDF	FTC	DRV/c	RAL	TAF	FTC	DRV/c	‐	Decreased renal function	38

ABC, abacavir; ART, antiretroviral therapy; DRV, darunavir; DTG, dolutegravir; EFZ, efavirenz; FTC, emtricitabine; TAF, tenofovir alafenamide fumarate; TDF, tenofovir disoproxil fumarate; RAL, raltegravir; 3TC, lamivudine; /c, cobicistat‐boosted; /r, ritonavir‐boosted.

* Same participant switches back to original regimen.

^†^ Switched back to initial regimen as the same adverse EVENT persisted.

## Discussion

In the RIVER trial, four‐drug ART regimens were well tolerated in this group of patients with PHI, reflected by the consistently high adherence levels and low occurrence of regimen switches. This was at a similar level to other three‐drug regimen cohorts [17] and despite a pill burden of four to six pills/day.

While only 15 participants (28%) had a truly ‘rapid’ ART start, everyone had commenced ART within four weeks of confirmed PHI diagnosis. Importantly, the design of the trial allowed the inclusion of those who had already started ART, and thus any delays should not have been attributed to screening/enrolment procedures for the trial. The RIVER trial patient population was small, male and highly motivated, limiting the generalizability to other cohorts, although despite this, our data demonstrate that four‐drug regimens are feasible in the PHI setting, including for rapid ART initiation.

While three‐drug regimens using a dolutegravir, bictegravir or darunavir/r third agent are recommended in PHI, barriers to four‐drug regimens (e.g. pill burden) are diminished with modern fixed‐dose combinations. Similarly, while the prevalence of transmitted INSTI resistance in the UK is currently low, this may rise with greater use [18]. However, it is acknowledged that the use of four‐drug combinations has become less common and is partially driven by physician choice dependent on the clinical circumstance (e.g. concerns about drug‐resistant HIV acquisition). It is also noted that studies comparing standard three‐drug regimens with five‐drug combinations that included raltegravir and maraviroc found no difference in viral suppression or HIV reservoir size, although sample sizes were small [19, 20].

Viable four‐drug regimens offer flexibility by expanding treatment options for people newly diagnosed with HIV, particularly in PHI, wishing to start treatment promptly. In these scenarios, clinical teams may also be reassured that a robust regimen is being utilized pending initial investigations; the regimen can then easily be rationalized when results are available or when viral suppression has been achieved.

## Author contributions

JEB and SLP drafted the initial manuscript. SLP developed the concept. WS performed the statistical analysis. All other authors contributed to the review and finalisation of the manuscript
